# Membranous nephropathy in a patient with coronavirus disease 2019 (COVID-19): A case report 

**DOI:** 10.5414/CNCS110379

**Published:** 2021-02-19

**Authors:** Jing Miao, Mary E. Fidler, Samih H. Nasr, Christopher P. Larsen, Ziad M. Zoghby

**Affiliations:** 1 Division of Nephrology and Hypertension,; 2 Department of Laboratory Medicine and Pathology, Mayo Clinic, Rochester, MN, and; 3 Arkana Laboratories, Little Rock, AR, USA

**Keywords:** acute kidney injury (AKI), coronavirus disease 2019 (COVID-19), membranous nephropathy (MN), renal pathology, SRAS-CoV-2

## Abstract

Introduction: Though respiratory, immune, and coagulation systems are major targets of coronavirus disease 2019 (COVID-19), kidney dysfunction, presenting with acute kidney injury (AKI), is also common. Most AKI cases in COVID-19 manifest as acute tubular injury (ATI) in conjunction with multiorgan failure. While initial renal pathological findings were limited to acute tubular necrosis and collapsing glomerulopathy, a recent case series reported a larger spectrum of findings. Case report: Here, we report a case of membranous nephropathy (MN) in an 81-year-old Hispanic man with underlying chronic kidney disease (CKD) stage 3 who developed ATI in the setting of COVID-19. The patient was hospitalized for hypoxic respiratory failure in the setting of AKI stage 3 with serum creatinine 7.1 mg/dL 6 days after a positive-SARS-CoV-2 screening. He was found to have nephrotic range proteinuria, glycosuria (with normal serum glucose), anemia, and hypoalbuminemia. Kidney biopsy showed ATI and early MN. Workup for primary and secondary MN was unrevealing, and serum PLA2R antibody was negative. No viral particles were observed in podocytes. Conclusion: Although the MN could be incidental, this observation raises the question of whether SARS-CoV-2 infection can trigger or worsen an underlying MN from an exaggerated immune response associated with COVID-19.

## Introduction 

Coronavirus disease 2019 (COVID-19), caused by a coronavirus named severe acute respiratory syndrome coronavirus 2 (SARS-CoV-2) has rapidly spread worldwide since December 2019 [1].The principle feature of COVID-19 is viral pneumonia, leading to acute respiratory distress syndrome (ARDS) [2]. Similar to other coronaviruses, angiotensin-converting enzyme 2 (ACE2) may play a major role in the entry of SARS-CoV-2 to its target cells [3]. Besides the respiratory system, ACE2 is also highly expressed in the brush border of proximal tubular cells and, to a lesser extent, in glomerular podocytes [4]. Kidney involvement of COVID-19, mainly presents as acute kidney injury (AKI) [5], primarily due to acute tubular injury (ATI) in the setting of multiorgan failure. Clinically, the incidence of AKI in COVID-19 varies from 0.9 to 29% in hospitalized or critically ill patients at different centers [6, 7, 8] and is associated with worse outcomes [5, 9]. ATI and direct parenchymal infection of tubular epithelial cells and podocytes were reported in 26 postmortem examinations of patients with severe COVID-19 [10]. Proteinuria and hematuria are also common, occurring in 44 and 27%, respectively [5]. Kidney biopsy findings have been reported initially in four living COVID-19 cases, all of which showed collapsing glomerulopathy [11, 12, 13, 14]. Recently, two case series of kidney biopsy findings showed that ATI was the most common finding in COVID-19-associated kidney injury, but the series by Kudose et al. [15, 16] reported a wide spectrum of glomerular and tubular disease including minimal change disease and membranous glomerulopathy. Here, we report a case of membranous nephropathy (MN) diagnosed in the setting of AKI associated with COVID-19. 

## Case report 

An 81-year-old Hispanic man presented to the emergency department complaining of progressive fatigue and shortness of breath 6 days after being diagnosed with COVID-19 (positive nasopharyngeal SARS-CoV-2 PCR). He reported myalgia, sore throat, intermittent dry cough, loss of smell and taste, poor appetite, and nausea without vomiting. He also had diarrhea and an episode of urinary incontinence. He denied fever or chills, chest pain, and headache. Because of hypoxemia requiring high-flow oxygen, the patient was admitted to the critical care unit. 

Previous medical history includes prostate cancer treated with chemotherapy and androgen deprivation therapy in 2013, in remission with undetectable prostate-specific antigen (PSA) since 2014, prediabetes, hyperlipidemia, hypertension, chronic kidney disease (CKD) stage 3 (baseline creatinine 1.2 – 1.6 mg/dL) attributed to hypertension with prior urine analysis in 2017 showing proteinuria of 385 mg/day, aortic valve stenosis, and cervical radiculopathy. Home medications included olmesartan 20 mg twice daily and hydrochlorothiazide 12.5 mg daily. Notably, he had a history of non-steroidal anti-inflammatory drugs (NSAIDs) use, 400 – 800 mg of ibuprofen per day for chronic neck pain. He is a former smoker but quit in 2012 and did not have lung disease. 

Initial vital signs: temperature 37.4 °C, blood pressure 166/69 mmHg, heart rate 68 beats per minute, respiratory rate 27 breaths per minute, and peripheral capillary oxygen saturation (SpO_2_) 95% on high-flow nasal cannula (50 L/min with FiO_2_ of 100%). Physical examination was notable for tachypnea with the remainder of physical examination unremarkable. 

Table 1 and Table 2 show his laboratory results. Repeat SARS-CoV-2 PCR via nasopharyngeal swab was positive. He had evidence of AKI stage 3 with a serum creatinine of 7.1 mg/dL. Urinalysis demonstrated 3 – 10 red blood cells per high power field, nephrotic range proteinuria of 4.6 g per 24 hours, glycosuria, and tubular epithelial cells. He had anemia with hemoglobin of 10.3 g/dL, hypoalbuminemia of 1.7 g/dL, and multiple elevated inflammatory markers, including interleukin-6 (IL-6), erythrocyte sedimentation rate (ESR), C-reactive protein (CRP), and ferritin. Kidney ultrasound revealed bilaterally increased parenchymal echogenicity consistent with CKD. Chest radiograph showed indistinct pulmonary vasculature bilaterally with bronchocentric ground glass, and bilateral patchy infiltrates, consistent with COVID-19 pneumonia. Lower extremities Doppler did not reveal deep vein thromboses, and transthoracic echocardiogram was normal. 

He was enrolled in a randomized placebo-controlled clinical trial of lenzilumab (monoclonal antibody targeting GM-CSF) 600 mg for 3 doses, in addition to intravenous antibiotics for possible superimposed community-acquired pneumonia. He completed a steroid trial with 5 days of intravenous methylprednisolone. Due to increasing D-dimer to 100,000 ng/mL, low-intensity heparin infusion was initiated. 

Over the first few hospitalization days, creatinine remained elevated at ~ 7 mg/dL. He did not require dialysis as he maintained excellent urine output. Serologic testing for hepatitis B, hepatitis C, human immunodeficiency virus (HIV), tuberculosis, as well as C3, C4, anti-neutrophil cytoplasmic antibodies (ANCA), anti-glomerular basement membrane (GBM), and anti-phospholipase A2 receptors (PLA2R) were all negative. Anti-THSD7A was indeterminate due to high background. No serum monoclonal proteins were detected. Kidney biopsy was performed on hospital day 4. 

On hospital day 7, the patient’s respiratory status worsened requiring intubation, mechanical ventilation, and initiation of vasopressors. Despite that, his AKI was slowly recovering and creatinine reached 3.7 mg/dL on hospital day 11. However, the shock state subsequently worsened, continuous dialysis was started but eventually the patient died on hospital day 16. Family declined an autopsy. 

## Kidney pathology findings 

**Figure 1.**
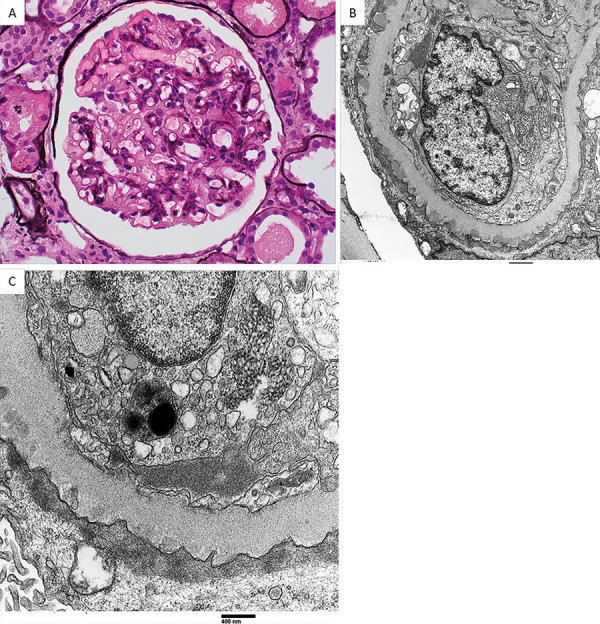
Renal pathologic findings in this COVID-19 patient. A: Glomerulus showing thickening of the glomerular basement membrane with mild mesangial sclerosis and hypercellularity (silver stain, × 400). B: Glomerular capillary loop showing abundant small sub-epithelial electron-dense deposits. The overlying podocytes show extensive foot process effacement (electron microscopy, × 11,000). C: Large glomerular endothelial tubuloreticular inclusion is shown. Tiny sub-epithelial electron-dense deposits are also evident (electron microscopy, × 30,000).

19 glomeruli were sampled for light microscopy, 8 of which were globally sclerotic and 1 was segmentally sclerotic. The glomeruli showed segmental mild mesangial hypercellularity and mesangial expansion, with thickening of their basement membranes (Figure 1A). No collapsing features, endocapillary hypercellularity, thrombi, necrosis, or crescents were seen. The tubulointerstitial compartment exhibited diffuse ATI, tubular protein reabsorption granules, mild tubular atrophy and interstitial fibrosis, and very mild mononuclear cell infiltrate without tubulitis. There was moderate arteriosclerosis and arteriolar hyalinosis. 

Immunofluorescence studies were unable to be performed due to an inadequate sample. Immunohistochemistry staining for PLA2R was performed on sections prepared from the paraffin block and was negative. 

In situ hybridization (ISH) staining for the presence of SARS-CoV-2 RNA was performed using RNAScope (ACD, Newark, CA, USA) and failed to show evidence of viral RNA in the kidney (methods in Supplemental Material). 

Electron microscopy showed abundant small granular sub-epithelial electron-dense deposits without or associated with early basement membrane spike formation (Figure 1B). The glomerular basement membrane lamina densa was thickened. There was mild mesangial sclerosis with segmental mesangial electron-dense deposits, without sub-endothelial deposits. Abundant tubuloreticular inclusion bodies were seen in the endothelial cell cytoplasm (Figure 1C). Podocytes exhibited severe foot process effacement. 

The pathological diagnosis was MN (stage 1 to early stage 2), diffuse ATI, mild mesangial sclerosing glomerulopathy (associated with hypertension, pre-diabetes, and smoking), and moderate arteriosclerosis and arteriolar hyalinosis. 

## Discussion 

To our knowledge, MN diagnosed in a patient with COVID-19 has been reported in only 2 patients in one case series of 17 patients [16]. The finding of severe ATI in this case is not surprising as reported in recent series of COVID-19 patients [10, 15]. However, the finding of MN was unexpected. 

MN, an inflammatory and autoimmune disease of the glomerulus, is one of the most common causes of nephrotic syndrome in adults. The etiology of ~ 75% of MN is unknown “primary”. Secondary MN can be secondary to infection, drugs, and malignancy [18]. Thus, the main question is whether the MN in this case is related to SARS-CoV-2 or not. This patient had a history of prostate cancer, but he was in remission for at least 6 years with normal PSA, so it is unlikely that his cancer was the underlying cause of the MN. NSAIDs can cause proteinuria and have been associated with minimal change disease as well as MN, but the patient did not have prior evidence of nephrotic syndrome despite being on NSAIDs for many years. The absence of detectable anti-PLA2R antibodies, the negative glomerular staining for PLA2R and the presence of mesangial deposits, as well as abundant tubuloreticular inclusions favor secondary MN over primary MN. The patient had mild proteinuria (385 mg/day) prior to COVID-19 which is likely due to underlying mild mesangial sclerosing glomerulopathy (associated with hypertension, prediabetes, and smoking). MN was mostly stage 1 favoring a recent development of disease temporally associated with COVID-19 over pre-existing MN. 

The pathogenesis of MN involves formation and deposition of immune complexes in sub-epithelial sites [18]. The receptor for SARS-CoV-2, ACE2, is highly expressed on proximal tubular cells and glomerular podocytes [4]. In addition, TMPRSS2, an essential serine protease, is required for spike glycoprotein of SARS-CoV-2 priming after binding to ACE2, and thus activates membrane fusion facilitating to gain access to its target cells [19]. In kidneys, expression of TMPRSS2 is only detectable in the proximal tubule S3 segment [20]. An in vitro study showed that the administration of TMPRSS2 inhibitor, camostat mesylate, had a valuable treatment effect, blocking multiple SARS-CoV-2 entry routes [21]. In postmortem kidney samples, SARS-CoV-2 antigens and viral particles were detected in the tubular epithelium and podocytes [10, 17]. In the case of collapsing glomerulopathy associated with COVID-19 reported by Kissling et al. [13], the virus was seen in podocytes by electron microscopy. However, most recent biopsy series fail to show viral particles in kidney biopsies by immunohistochemistry staining or by electron microscopy arguing against a direct viral infection of the kidneys [15, 16]. Similarly, in this case we did not find evidence of viral particles in the kidneys. Rather than a direct toxic viral effect on the kidneys, the ATI is most likely cytokine mediated, although the NSAID, angiotensin receptor blocker, and diuretic exposures could also have contributed. Whether MN can be secondary to SARS-CoV-2 remains to be elucidated, but we speculate that it could result from an exaggerated immune response associated with COVID-19. In the passive Heymann nephritis model, sub-epithelial deposits with very early basement membrane reaction could be seen as early as 7 days after injection [22]. Therefore, we hypothesize that the development of MN deposits could possibly occur quickly after a viral infection, or alternatively the COVID-19-related immune response and the resulting high-grade proteinuria could unmask an underlying MN. If this is the case, the treatment of this patient’s MN is conservative and immunosuppressive therapy is not recommended. 

## Funding 

None. 

## Conflict of interest 

The authors declare no relevant financial interest. 

## Supplemental material 

### ISH methods 

In situ hybridization was performed with RNAScope (ACD, Newark, CA) using probes directed against SARS-CoV-2 on formalin-fixed paraffin-embedded tissue sections cut at a thickness of 3 microns. ^1^A negative control (bacterial gene dapB) was also included to assess background signals as well as positive control probes to the housekeeping gene peptidylprolyl isomerase B (*PPIB*). The ISH sections were counterstained using periodic acid-Schiff. (Wang F, Flanagan J, Su N, Wang LC, Bui S, Nielson A, et al. RNAscope: a novel in situ RNA analysis platform for formalin-fixed, paraffin-embedded tissues. J Mol Diagn. 2012; *14:* 22-29). 

**Table 1.** Laboratory data.

Laboratory test	1 year before admission	Day 1	Day 4 (Kidney biopsy)	Day 7	Reference
Arterial blood gas					
pH		7.43	7.36	7.29	7.35 – 7.45
pCO_2_, mmHg		23	27	38	32 – 45
pO_2_, mmHg		137	68	72	83 – 108
HCO_3_^-^, mmol/L		15	15	18	22 – 26
Complete blood count					
WBC count, 10^9^/L		11.2	15.0	15.1	3.4 – 9.6
Neutrophils, 10^9^/L		10.35			1.56 – 6.45
Lymphocytes, 10^9^/L		0.46			0.95 – 3.07
Erythrocytes, 10^12^/L		3.37	3.40	2.36	4.35 – 5.65
Hemoglobin, g/dL		11.3	11.4	7.9	13.2 – 16.6
Reticulocytes, %				2.12	0.6 – 2.71
Platelet count, 10^9^/L		449	436	295	135 – 317
Serum biochemistry					
Sodium, mmol/L		138	139	138	135 – 145
Potassium, mmol/L		5.4	4.5	4.1	3.6 – 5.2
Chloride, mmol/L		100	99	103	98 – 107
Bicarbonate, mmol/L		17	17	19	22 – 29
Anion gap		21	23	17	7 – 15
BUN, mg/dL		98	133	128	8 – 24
Creatinine, mg/dL	1.4	7.05	6.96	4.41	0.74 – 1.35
eGFR, mL/min/BSA	49	< 15	< 15	< 15	> 60
eGFR by cystatin C, mL/min/BSA			6		> 60
Calcium, total, mg/dL		8.9	8.3	8.1	8.8 – 10.2
Calcium, ionized, mg/dL		4.40	4.57	4.62	4.65 – 5.30
Glucose, mg/dL		127	165	145	70 – 140
Magnesium, mg/dL			3.8	2.9	1.7 – 2.3
Phosphorus, mg/dL			11.5	7.9	2.5 – 4.5
Total protein, g/dL		4.4		4.5	6.3 – 7.9
Albumin, g/dL		1.7		2.6	3.5 – 5.0
Hemoglobin A1_C_, %	5.9				4 – 5.6
Lactate, mmol/L		1.3		1.1	0.5 – 2.2
Liver function					
ALT, U/L	45	36		11	7 – 55
AST, U/L	43	49		22	8 – 48
Bilirubin, total, mg/dL	0.6	< 0.2		0.5	< 1.2
Bilirubin, direct, mg/dL	0.3	< 0.2		0.4	0.0 – 0.3
Alkaline protease, U/L		155		70	40 – 129
Lipid/cardiac risk					
Total cholesterol, mg/dL	241		226		< 200
HDL, mg/dL	38		34		≥ 40
LDL, mg/dL	153		131		< 100
Triglycerides, mg/dL	248		304		< 150
Troponin T, ng/L		71		160	< 15
Troponin T-2h, ng/L		80		153	< 15
Troponin T-6h, ng/L		87		152	< 15
NT-pro BNP, pg/mL	220	5,030			5 – 131
Creatinine kinase, U/L				108	39 – 308
Coagulation					
Antithrombin activity			91		80 – 130%
D-dimer, ng/mL		13,286	> 100,000	48,550	< 500
Fibrinogen, Clauss, mg/dL			> 800	561	200 – 500
Coag factor II			101	92	75 – 145%
Coag factor V			132	110	70 – 165%
Coag factor VII			116	83	65 – 180%
Coag factor X			131	86	70 – 150%
C-reactive protein, mg/L		> 400	173.3	142.1	< 8
Soluble fibrin monomer, mcg/mL			> 1,100	36	≤ 8
Plasminogen activity			98		75 – 140%
α-2 plasmin inhibitor			105		80 – 140%
Sedimentation rate, mm/h		> 140	123		3 – 28
Ferritin, µg/L		1,122	1,813	911	24 – 336
Serology					
HBs antigen		Negative			Negative
HBc total Ab		Negative			Negative
HCV Ab screen		Negative			Negative
HIV-1/-2 Ag and Ab		Negative			Negative
Complement C3, mg/dL		163			75 – 175
Complement C4, mg/dL		36			13 – 40
C-ANCA			Negative		Negative
p-ANCA			Negative		Negative
Anti-GBM, U			< 0.2		< 1 (negative)
Anti-phospholipase A2 receptor (IF)			Negative		Negative
Anti-phospholipase A2 receptor (ELISA), RU/mL			< 2		< 14
Interleukin 6, pg/mL		39.5	3.5	5.7	< 1.8
Monoclonal gammopathy screen					
κ free light chain, mg/dL			15.5		0.33 – 1.94
λ free light chain, mg/dL			8.73		0.57 – 2.63
κ/λ ratio			1.78		0.26 – 1.65
Total protein, g/dL			5.3		6.3 – 7.9
Albumin, g/dL			1.4		3.4 – 4.7
α-1 globulin, g/dL			0.6		0.1 – 0.3
α 2-globulin, g/dL			1.5		0.6 – 1.0
β globulin, g/dL			1.0		0.7 – 1.2
γ globulin, g/dL			0.8		0.6 – 1.6
A/G ratio			0.36		
M protein isotype			Cannot rule out small monoclonal protein		
Endocrine					
TSH, mIU/L	1.1			0.2	0.3 – 4.2
T4 (thyroxine), ng/dL				1.3	0.9 – 1.7
PTH, pg/mL		231			15 – 65
Tumor/malignancy marker					
Prostate specific Ag, ng/mL	0.21	0.15			≤ 7.2

**Table 2.** Urinalysis data.

Laboratory test	3 years prior to admission	On admission	Day 2	Reference range
Source	Midstream	Catheter	Catheter	
Appearance	Normal	Normal	Normal	
Osmolality, mOsm/kg		372	339	150 – 1150
pH		5.2	5.5	4.5 – 8.0
Glucose, mg/dL	5	81	12	0 – 15
Protein, mg/dL	17	339	117	< 26
Protein/Osmolality, ratio	0.39	9.11	3.45	< 0.42
Predicted 24 h protein, mg	385	7,735	3,066	
24-h urine protein, mg/24 h			4,662	< 229
Hemoglobin	Negative	Trace	Moderate	Negative
Red blood cell			3 – 10	< 3/HPF
Dysmorphic RBC (%)			< 25	< 25
White blood cell		1 – 3	1 – 3	1 – 3/HPF
Casts, hyaline	1 – 3		Occasional	
Casts, granular			Occasional	
Fat, free		Occasional	Occasional	
Fat, in casts			Occasional	
Oval fat body			Occasional	
Renal epithelial cells		1 – 3		None seen/HPF
Ketones		Negative		Negative
Nitrite		Negative		Negative
Leukocyte		Negative		Negative
